# Comparative Proteomic Analysis Revealed the Mechanism of Tea Tree Oil Targeting Lipid Metabolism and Antioxidant System to Protect Hepatopancreatic Health in *Macrobrachium rosenbergii*

**DOI:** 10.3389/fimmu.2022.906435

**Published:** 2022-05-31

**Authors:** Mingyang Liu, Cunxin Sun, Xiaochuan Zheng, Qunlan Zhou, Bo Liu, Yifan Zhou, Pao Xu, Bo Liu

**Affiliations:** ^1^ Wuxi Fisheries College, Nanjing Agricultural University, Wuxi, China; ^2^ Key Laboratory of Aquatic Animal Nutrition and Health, Freshwater Fisheries Research Center, Chinese Academy of Fishery Science, Wuxi, China; ^3^ Key Laboratory of Freshwater Fisheries and Germplasm Resources Utilization, Ministry of Agriculture and Rural Affairs, Freshwater Fisheries Research Center, Chinese Academy of Fishery Sciences, Wuxi, China; ^4^ College of Fisheries and Life Sciences, Shanghai Ocean University, Shanghai, China

**Keywords:** *Macrobrachium rosenbergii*, tea tree oil, lipid metabolism, antioxidant capacity, hepatopancreatic health

## Abstract

Tea tree oil (TTO) is a pure natural plant essential oil. The studies evaluated the hepatopancreas lipid metabolism and antioxidant efficacy of *Macrobrachium rosenbergii* fed with 0 (CT group) and 100 mg/kg TTO (TT group) by label-free quantification proteomic analysis. Compared to the CT group, the TT group improved growth performance and increased the survival rate after stress. Dietary TTO also decreased hemolymph AST and ALT activities and decreased hepatopancreatic vacuolation. At the same time, hepatopancreas lipids droplets and hemolymph lipids (TG, TC, LDL-C) were decreased, and the peroxidation products content (MDA, LPO, 4-HNE) was also decreased. In addition, the levels of hepatopancreas antioxidant enzymes (T-AOC, CAT, and SOD) were increased in the TT group. With proteomic analysis, a total of 151 differentially expressed proteins (DEPs) (99 up-regulated and 52 down-regulated) were identified in the hepatopancreas. Kyoto Encyclopedia of Genes and Genomes (KEGG) and protein-protein interaction analysis showed that the 16 DEPs have interactions, which are mainly involved in the pathways related to lipid metabolism (fatty acid biosynthesis, fatty acid metabolism, glycerophospholipid metabolism) and redox reaction (cytochrome P450 enzyme systems). Furthermore, the mRNA expression of 15 proteins followed the proteomic analysis with qRT-PCR validation. Pearson correlation analysis showed that fatty acids and glycerophospholipid metabolism-related proteins were highly correlated to peroxide content, glycerophospholipid metabolism, and cytochrome P450 system-related proteins (CYP1A1, GSTT1, GPX4) were highly correlated to AST and ALT. Additionally, GPX4 is closely related to peroxide content and antioxidant enzyme activity. Our results revealed that TTO plays a protective role in the hepatopancreas targeting the critical enzymes and antioxidant reactions in lipid metabolism. Provides a new perspective to elucidate the action path of TTO in protecting invertebrate hepatopancreas, highlights the influence of lipid metabolism on hepatopancreas health and the interaction between lipid metabolism and antioxidant system in the regulation of TTO.

## Introduction

Plant essential oils were extracted from plants and have been widely used as antioxidants in the aquaculture industry in recent years (1). Previous studies demonstrated that dietary plant essential oils had a beneficial effect on animal health as their inherent bioactive constituents with antioxidant, anti-stress, growth-promoting, and immune-stimulating properties (2, 3). Tea tree oil (TTO) was used as essential plant oil and obtained by steam distillation of branches and leaves on *Melaleuca alternifolia* (4). Studies have shown that TTO has more effective antibacterial activity than other essential oils (5). At the same time, TTO was one of the promising antioxidants in aquaculture. Our previous study showed that an appropriate level of TTO could significantly improve the growth performance of *Macrobrachium rosenbergii* without negative effects (6). Meanwhile, we also found that TTO could reduce the mortality of prawns, activate several non-specific immune factors and ROS systems by triggering the NF-κB signaling pathway in prawns (7). However, the specific pathways that affect anti-oxidation remain to be further studied.

Lipid is an essential component of living organisms and the primary energy supply material. In crustaceans, lipids were mainly deposited in the hepatopancreas, which played an essential role in lipid metabolism and the immune system (8). Meanwhile, crustaceans are very sensitive to diet lipid content. For example, high lipid could lead to metabolism disorders of the hepatopancreas or destroy the antioxidant balance. Hepatopancreas lipid metabolism disorders could affect physiological functions and cause immune system damage (9). In addition, fat hyperdeposition and peroxidation caused by lipid metabolism disorders could cause oxidative stress and damage the antioxidant system (10). Some studies found that plant essential oils could reduce the AST and ALT activity and the lipid deposition in the hepatopancreas, not only to aquatic animals but also to poultry (11, 12). However, the mutual regulations among lipid metabolism, antioxidant pathways and the action mechanism of essential oils have still not been clarified.

In recent years, the availability of proteomics sequencing has facilitated a new analytical approach for revealing the translation status of proteins and the regulation of translation expression at the whole level (13). The label-free quantification proteomic approach allows simultaneous identification and quantification and applies to samples from any source (14–16). Existing studies have used proteomics sequencing to screen the differentially expressed proteins (DEPs) in the hepatopancreas and then explore the interrelationships among DEPs on *M. rosenbergii* and *Eriocheir sinensis* after feed additives intervention (17, 18), which demonstrates the feasibility of this technique in spineless animals.

The giant river prawn, *M.rosenbergii*, was farmed in China on a large scale, initially introduced into China from Thailand in 1976 (19). Because of their beneficial characteristics, including fast-growing rate, high resistance to biotic and abiotic stress, and available genomic annotation information, *M. rosenbergii* also served as an important research model for immunology, nutrition, and metabolism in crustaceans (20, 21). Lipid is an important substrate for nutrients in the crustaceans. The hepatopancreas is main function of lipid metabolism for energy supply and the key organs of immunity. The hepatopancreas was used as a model organ for proteomics analysis to study the effect of TTO on the lipid metabolism and antioxidant pathway of *M. rosenbergii*. In this study, the lipid droplets of hepatopancreas were determined by oil red O staining. Then, the label-free quantification proteomic approach was used to analyze protein expression and identify the differentially expressed proteins in the hepatopancreas of the prawns fed with 100 mg/kg TTO and control diets.

## Materials and Methods

### Experimental Materials, Design, and Growth Evaluation

All experiments were performed following the guidelines on the care and use of animals for scientific purposes set by the Institutional Animal Care and Use Committee (IACUC) of the Chinese Academy of Fishery Science (CAFS). *M. rosenbergii* was obtained from Zhejiang South Taihu Lake Freshwater Seeds Co., Ltd. (Huzhou, China). Prawns of similar size were acclimated in dechlorinated tap water under laboratory conditions (temperature, 29-32°C; pH, 7.5-8.4; dissolved oxygen, 6± 0.43 mg/L) for 7 days. Before the feeding experiment, a total of 300 prawns (average initial weight, 0.39 ± 0.01 g) were separated into 6 cement tanks (L×W×H, 2.0 m × 1.5 m × 0.5 m) randomly at 50 prawns per tank. The prawns were fed with commercial feed (41% crude protein, 7% crude lipid) during the acclimation period. 6 tanks were randomly assigned to two experimental diet groups with 3 replicates. The detailed formulation and proximate composition of the diets were described in **Table 1**. The prawns were fed three times a day, at 8:00 am 1:00 pm, and 6:00 pm. After an 8-week feeding experiment, we recorded the weight of prawns in each group. Then according to the Tadese et al. method (12), we took out 20 prawns from each tank for 24h ammonia nitrogen stress experiment, and the survival rate of prawns in each tank was recorded after 24h. The weight gain rate, specific growth rate, feed intake, feed conversion ratio, hepatopancreas index, condition factor, and survival rate after stress were calculated in the following methods:

**Table 1:** The formulation and proximate composition of the experimental diet.

Ingredients (g kg^-1^)	CT	TT
Fish meal^1^	350.00	350.00
Soybean meal^2^	100.00	100.00
Rapeseed meal^2^	100.00	100.00
Shrimp meal^1^	80.00	80.00
Squid extract^1^	30.00	30.00
Soybean oil^2^	80.00	80.00
Fish oil^2^	80.00	80.00
α-starch^1^	120.00	119.00
Lecithin powder^1^	10.00	10.00
Choleserol^1^	3.00	3.00
Ecdysone^1^	2.00	2.00
MCP	20.00	20.00
Premix^3^	10.00	10.00
Choline chloride^4^	10.00	10.00
Bentonite^4^	5.00	5.00
10% Tea Tree Oil	0.00	1.00
Total	1000	1000
Proximate analysis (%)		
Moisture	10.40	9.48
Crude protein	40.91	39.46
Crude lipid	10.76	10.06
Ash	17.43	16.52

^1^ Obtained from Jiangsu Fuyuda Food Products Co., Ltd., Yangzhou, China; ^2^Obtained from Hulunbeier Sanyuan Milk Co., Ltd., Inner Mongolia, China;^3^Premix supplied the following minerals (g kg^-1^) and vitamins (IU or mg kg^-1^):CuSO_4_·5H_2_O, 2.0 g; FeSO_4_·7H_2_O, 25 g; ZnSO_4_·7H_2_O, 22 g; MnSO_4_·4H_2_O, 7 g; Na_2_SeO_3_, 0.04 g; KI, 0.026 g; CoCl_2_·6H_2_O, 0.1 g; Vitamin A, 900,000 IU; Vitamin D, 200,000 IU; Vitamin E, 4500 mg; Vitamin K_3_, 220 mg; Vitamin B_1_, 320 mg; Vitamin B_2_, 1090 mg; Vitamin B_5_, 2000 mg; Vitamin B_6_, 500 mg; Vitamin B_12_, 1.6 mg; Vitamin C, 5000 mg; Pantothenate, 1000 mg; Folic acid, 165 mg. Vitamin and mineral additives were provided by Wuxi Hanove Animal Health Products Co., Ltd.^4^ Obtained from Freshwater Fisheries Research Center, Chinese Academy of Fishery Sciences.

Weight gain rate (WGR, %) = (Final weight − Initial weight)/initial weight × 100

Specific growth rate (SGR, %day^−1^) = (Ln final weight − Ln initial weight) × 100/days

Feed intake (FI, % body weight days^−1^) = 100 × feed consumption/[(initial weight + final weight)/2]/days

Feed conversion ratio (FCR) = Dry feed intake (g)/weight gain (g)

Hepatopancreas index (HSI, %) = (Hepatopancreas weight/final weight) × 100

Condition factor (CF, g/cm^3^) = 100 × [body weight/body length^3^ (cm^3^)]

Survival rate after stress (SR, %) = 100 × final number of prawns/20

### Samples Collection, Biochemical Analysis, and Enzyme Assays

At the end of the feeding trial, all the prawns fasted for 24 h. The hepatopancreas samples of 9 prawns in each group were randomly sampled and stored at -80°C for proteomic analysis. Then, hemolymph samples were extracted from the cardiocoelom of 9 prawns in each group (total 18 prawns) and centrifuged at 4000 rpm for 10 min at 4°C for separation of hemolymph from blood cells (22). Then, the hepatopancreas was collected from 18 prawns in sterile centrifuge tubes and immediately frozen in liquid nitrogen to further detect enzyme activity. The hepatopancreas for enzyme activity test was homogenized in ice-cold physiological saline (0.9%w/v NaCl) and centrifuged at 3500 rpm for 25 min at 4°C (Eppendorf Scientific 5810R centrifuge), with the resulting supernatants aliquoted and kept at -20°C until the subsequent analysis. Additionally, the hepatopancreas of another 18 prawns (9 samples per group) was selected and placed in a centrifuge tube filled with 1 ml of TRIzol for total RNA extraction. Finally, the hepatopancreas of 6 shrimps (3 per group) was sampled and placed in 4% buffered paraformaldehyde (Biosharp, Labgic, Technology Co., LTD, Hefei, China) for oil red O staining and H&E staining.

Hemolymph concentrations of triacylglycerol (TG), total cholesterol (TC), low-density lipoprotein cholesterol (LDL-C), high-density lipoprotein cholesterol (HDL-C), and enzymatic activity of aspartate transaminase (AST), alanine transaminase (ALT) were determined using commercial kits (Mindray BS-400, Shenzhen, China) following the manufacturer’s instructions (23). The measurement of all the indices was carried out using an automatic biochemical analyser (Mindray, BS-400, Shenzhen, China).

The activities of superoxide dismutase (SOD), catalase (CAT), and total antioxidant capacity (T-AOC) in the hepatopancreas were measured using kits from Nanjing Jiancheng Bioengineering Institute. Content of Lipid peroxidation (LPO) and 4-hydroxynonenal (4-HNE) were determined by ELISA kits specific for *M. rosenbergii* (Shanghai mlbio biotechnology Co., Ltd., China) according to the manufacturer’s instructions. Tissues homogenates were performed before enzyme analyses. Tissues involving hepatopancreas were suspended in 50 mM potassium phosphate buffer with 0.5 mM EDTA at pH of about 7.0. Each of the homogenization tubes was then added with the 50 microlitres of Triton X-100 and a few crystals of phenylmethylsulfonyl fluoride. Homogenates of the hepatopancreas tissues were performed in microcentrifuge tubes using a plastic hand pestle for 5 min. At the termination of the homogenization process, all-inclusive samples were subjected to centrifuge set at 13,000 rpm for 15 min at 2°C. The supernatant resultant was removed for use in enzyme analyses.The all-inclusive enzyme activity analyses were conducted using a Spectra Max Plus 384 spectrophotometer (Molecular Devices, Menlo Park, CA, USA). Malonaldehyde (MDA) content in the hepatopancreas was estimated using the thiobarbituric acid (TBA) test (24) by the assay kit from Nanjing Jiancheng Bioengineering Institute, China. MDA assay method is the same as enzyme.

### H & E and Oil Red O Staining

Samples were embedded in Optimal Cutting Temperature (OCT) medium and stored at -80°C. OCT embedded, 7 μm sections were stained with Oil Red O (ORO) (Sigma-Aldrich, St. Louis, MO, USA) for fat content examination. Randomly chosen tissue sections were photographed using an Olympus BX51 light microscope (Tokyo, Japan) equipped with Olympus UPlan Apo 200× objective. The ORO-stained sections were analyzed for lipid content by the ImageJ Software (National Institute of Mental Health, USA) adapted for ORO-stained sections algorithm and expressed as % of hepatopancreas tissue area stained positively for lipids, and finally determine its gray value. H & E staining hepatopancreas structural section of the hepatopancreas was conducted following the method detailed by Liu et al. (6).

### Protein Preparation and Labeling

The hepatopancreas samples were ground into a powder with liquid nitrogen for protein extraction, and hepatopancreas protein was extracted using a total protein extraction kit (Solarbio Biotechnology, Co., Ltd., Beijing, China). The protein supernatant was separated by high-speed centrifugation at 12,000 ×g for 10 min at 4°C. The protein concentration of the supernatant was determined using a bicinchoninic acid (BCA) kit (Beyotime Biotechnology, Co., Ltd., Shanghai, China), following the manufacturer’s instructions. For protein digestion, 100 μg of protein supernatant was first reduced with 5 mM dithiothreitol at 56°C for 30 min and then alkylated with 11 mM iodoacetamide at room temperature for15 min in the dark. Next, the digestion mixture was diluted with100 mM triethylammonium bicarbonate (TEAB) to reduce urea concentration to less than 2 M. Trypsin was added at a ratio of 1:50 and the digestion mixture was incubated at 37°C overnight, followed by the addition of trypsin at a ratio of 1:100 and incubation for 4 h for complete digestion. After digestion, the peptides were desalted using Strata X C18SPE column (Phenomenex) and dried under vacuum. Next, the dried peptides were reconstituted in 100 mM TEAB and labeled with a 0.8 mg10-plex TMT label according to the manufacturer’s protocol.

### Liquid Chromatography-Tandem Mass Spectrometry Analysis

The labeled peptides were pooled and fractionated using a high-performance liquid chromatography (HPLC) system equipped with high pH reverse-phase Agilent 300 Extend C18 column. A total of 18 fractions were generated using a gradient of 8–32% acetonitrile over 60 min. LC-MS/MS analysis was conducted for 40 min for each fraction using the EASY-nLC 1000 UPLC system coupled with Thermo Scientific Q Exactive Plus to separate and identify complex peptides. Fractions were reconstituted in 0.1% formic acid (FA) and loaded onto a reversed-phase analytical column (Acclaim Pep Map^®^ RSLC C18, 2 μm,100 A, 75 μm × 15 cm) at a constant flow rate of 400 nL/min. Full-MSspectra were acquired using an Orbitrap analyzer at a resolution of 70,000. Ion fragments were detected in the Orbitrap at a resolution of 17,500. The 20 most intense precursors were selected for subsequent HCD fragmentation at a collision energy of 28 and dynamic exclusion for 15s. The resulting raw data were searched against the *M. rosenbergii* Uniprot database (27,789 sequences) with reverse decoy database using Max quant search engine (v.1.5.2.8). Trypsin/P was selected as the cleavage enzyme, and up to 2 missed cleavages were allowed. Carbamidomethylation (C), TMT 10plex (N-term), and TMT 10plex (K) were set as fixed modifications and oxidation (M) was set as variable modification. Precursor mass tolerance was set to 20 ppm, and fragment deviation was set to 0.02 Da. Peptide global false discovery rate (FDR) was adjusted to < 1%, and the minimum peptide score was set > 40 (25).

### Bioinformatic Analysis

In this study, bioinformatic analysis of the proteome was performed. For the proteome analysis, the assembled unigenes were aligned with the non-redundant database (Nr), Gene Ontology (GO), eukaryotic ortholog Groups (KOG), Kyoto Encyclopedia of Genes and Genomes (KEGG) by using blast to obtain annotation of the genes. Cluster enrichment of GO terms and KEGG pathways were analyzed using the hypergeometric distribution. TBtools v1.09854 analyzed the heatmap by uploading the proteins id and absolute quantification of protein. The color selection was red and blue, the clustering method was average linkage, and the distance measurement method was Pearson. Protein-protein interaction (PPI) network was obtained from the STRING database setting at medium confidence (0.4), the organism chose *Drosophila melanogaster*. Then protein expression data and KEGG pathway annotation data were integrated into the network for visualization using Cytoscape 3.8.2 (26).

### RNA Extraction and RT-PCR Analysis

Primer 5.0 was used to design primers for genes for qRT-PCR analysis based on the obtained fragments from the original transcriptome (shown in **Table 2**). According to the reference of Tadese et al. (12), because of the stable expression of β-actin, β-actin mRNA was chosen as an internal reference. The PCR primers were produced by Shanghai Sangon Biotechnology, Co., Ltd., China.

**Table 2:** Primers and sequences in the experiments.

Gene	Sequences (5’-3’)	Product length (bp)	Tm (°C)
*β-actin*	(F)TCCGTAAGGACCTGTATGCC (R)TCGGGAGGTGCGATGATTTT	198	59.97
			59.69
*ACOX1*	(F)GACTTCCGCACTAACCCACT (R)AGCTTCAGGACTGTTCCACC	116	60.04
			59.97
*ACOX3*	(F)ACGACATTGGATGCTTTGGC (R)CTTCTGTGCCAAGGGGTGAG	171	60.39
			59.61
*ACACA*	(F)GCAGATCAAGTTCGAGGGCT (R)TGTTGTGCCGTTGGTTTGTG	186	59.05
			59.56
*ECHS1*	(F)ATGCCCTTTCACTACAAATGCTG (R)AAGACCTTGCCTTGAGCACC	134	60.00
			58.23
*FASN*	(F)TGGTGATCTTCCTCCAGCGT (R)ACGTAGGACACATCTGAGGGA	147	60.18
			59.40
*ATP5B*	(F)CTCCAAGGGAAGGGTTGCTA (R)AGGTCTGGCAAGGGAGAATC	134	60.27
			60.20
AGPAT4	(F)TTGTCGAAGTGCTGGTCTGT (R)CTGGACGGCAATGAATCCCT	227	59.41
			59.52
LPGAT1	(F)CCATCTCGTCCGTATGTCCC (R)AACCCTCGATCCACTCCTGA	156	59.00
			58.93
ACHE	(F)GAAACTCGAGCCGGCGAA (R)CGACGGTGATGTTGTTGCC	192	59.38
			59.40
Gpdh1	(F)GTCCAGTCCTCCTGTACTCCTC (R)TTAAGAGCAGAGCCGCAATCA	106	59.79
			59.96
CYP1A1	(F)GCAGTTTGCACAGACGTGTT (R)CCATGCTGGGAAAACGATTA	152	60.05
			60.06
CYP15A1	(F)GATGGCGTAGTAGCGGTCAA (R)GCCGCTGGCTCTTCAGTT	179	60.01
			59.73
GSTT1	(F)ATGTCCAGTCCTCCTGTACTCC (R)AAGAGCAGAGCCGCAATCAG	206	59.18
			58.87
GPX3	(F)TCTGTTACCGCTGGTGGCTG (R)TGTGAACCGTTTAGTGTTTTGGC	184	59.28
			59.72
GPX4	(F)GACTTCGTGCTGGTCGCTT (R)ATTCGCCTCGTCGTAGCC	107	59.98
			59.65
HSP70	(F)CTGGATGACACTACTCGGGAAG (R)CCTCATTGTCGTTGTAGTCGTG	164	59.26
			59.50

The mRNA sequences for each gene were obtained from M. rosenbergii hepatopancreas transcriptome sequencing database of aquatic disease and feed laboratory of Freshwater Fisheries Research Center, Chinese Academy of Fishery Sciences. Primers for RT-PCR were designed using Primer premier 5.0. ACOX1, acyl-CoA oxidase 1; ACOX3, acyl-CoA oxidase 3; ACACA, acetyl-CoA carboxylase; ECHS1, enoyl-CoA hydratase 1; FASN, fatty acid synthase; ATP5B, ATP synthase subunit beta 5; AGPAT4, Acylglycerophosphate acyltransferase 4; LPGAT1, lysophosphatidylglycerol acyltransferase 1; ACHE, Acetylcholinesterase; Gpdh1, glycerol-3-phosphate dehydrogenase 1; CYP1A1, cytochrome P4501A1; CYP15A1, cytochrome P45015A1; GSTT1, glutathione S-transferase-theta 1; GPX3, Glutathione peroxidase 3; GPX4, glutathione peroxidase 4; HSP70, heat shock 70kDa protein.

Total RNA of the hepatopancreas in the two groups (9 samples per group) were extracted by RNAiso Plus (TaKaRa, Japan) and measured by Nanodrop 2000 (Thermo Fisher Scientific, USA). The RNA in each sample was diluted to 500ng/ml, and the following quantitative analysis was carried out for 2 μg of the total RNA with a Two Steps SYBR^®^ Prime Script^®^ Plus RT-PCR Kit (TaKaRa, Dalian). The Real-time quantitative PCR (RT-PCR) was used based on Liu et al. (27) and conducted by ABI 7500 real-time PCR system, and the2-^△△CT^ method was used for analysis.

### Date Analysis

Data first deriving from each feeding experiment were subjected to the standard distribution test and homogeneity of variance with Levene’s test. Independent-samples T-test was used on the result by SPSS (version 25). Pearson correlation analysis was used to analyze the correlation between two variables that conform to normality. In the test, one asterisk (*) indicates a significant difference (*P* < 0.05), two asterisks (**) indicate an extremely significant difference (*P* < 0.01). All the results were expressed as the mean ± standard error of the mean (X ± SEM).

## Results

### Growth Evaluation

Growth performances of the prawns are presented in **Table 3**. The prawns that were fed with 100 mg/kg TTO diets displayed significantly improved growth performance, including WGR, SGR, FI and CF (*P* < 0.05). Simultaneously, the TT group significantly decreased HSI compared to the CT group (*P* < 0.05). The TT group has better FCR and SR in the feeding trial but showed no difference to the CT group (*P* > 0.05). After ammonia stress, the SR of the TT group was significantly higher than that of the CT group (*P* < 0.05).

**Table 3:** Effects of dietary TTO on growth evaluation of *M. rosenbergii*.

Index	Groups	
	CT	TT
Initial weight (g)	0.38 ± 0.01	0.39 ± 0.01
Weight gain rate (%)	3437.67 ± 78.32^a^	4165.31 ± 65.14^b^
Specific growth rate (%/day^-1^)	4.04 ± 0.12^a^	4.82 ± 0.03^b^
Feed intake (% body weight day^-1^)	3.92 ± 0.26^a^	4.73 ± 0.34^b^
Feed conversion ratio	1.45 ± 0.06	1.38 ± 0.0.4
Hepatopancreas index (%)	7.03 ± 1.06^b^	5.93 ± 0.86^a^
Condition factor (g/cm^3^)	2.24 ± 0.06^a^	3.50 ± 0.86^b^
Survival rate in feeding trial (%)	87.50 ± 2.67	91.33 ± 4.37
Survival rate after stress (%)	30.00 ± 5.77^a^	43.33 ± 3.33^b^

Data are expressed as means with SEM. Value with different superscripts is significantly different (P < 0.05).

### Hepatopancreatic Health Status Analysis

**Figure 1:**
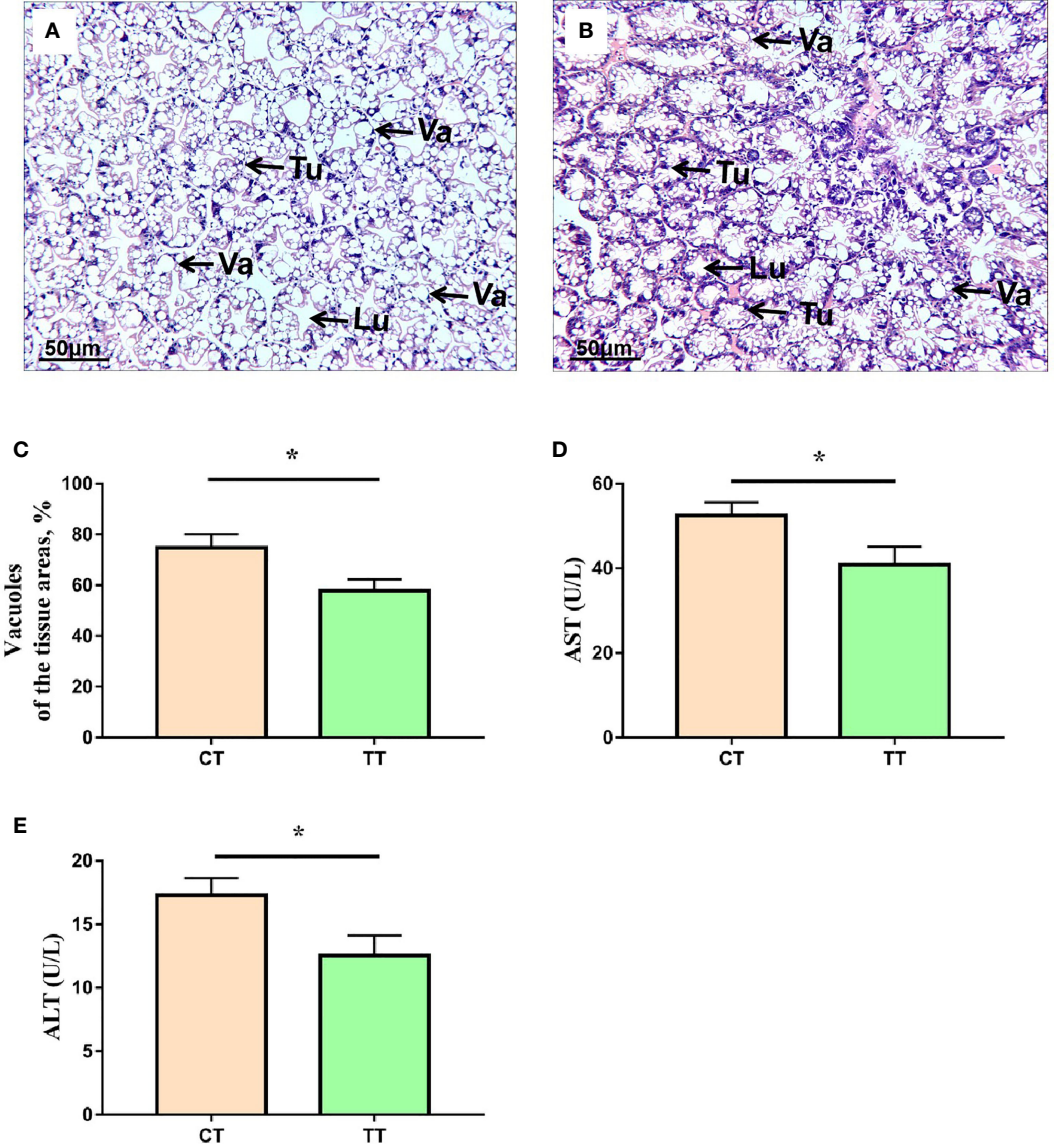
Effects of TTO diet on morphology and function in hepatopancreas tissues of *M. rosenbergii*. **(A)** CT group H & E stainings; **(B)** TT H & E stainings. Tu, tubule; VA, vacuole; Lu, lumen. **(C)** Vacuoles areas; **(D)** Aspartate transaminase (AST); **(E)** Alanine transaminase (ALT). Data are expressed as means with SEM. Value with an asterisk is significantly different (P < 0.05).

To evaluate the health status of hepatopancreas, we performed histological analysis and hemolymph biochemical analysis. H & E staining results showed that the TT supplementation reduced the number of vacuoles and obvious cell boundaries (**Figures 1A–C**). The CT group have less tubules, swollen lumen and unclear boundaries in the hepatopancreas (**Figures 1A, B**). The levels of AST and ALT activities in hemolymph were significantly reduced in the TT group compared to the CT group (*P* < 0.05; **Figures 1D, E**).

### Hepatopancreas Lipid Droplets Content

**Figure 2:**
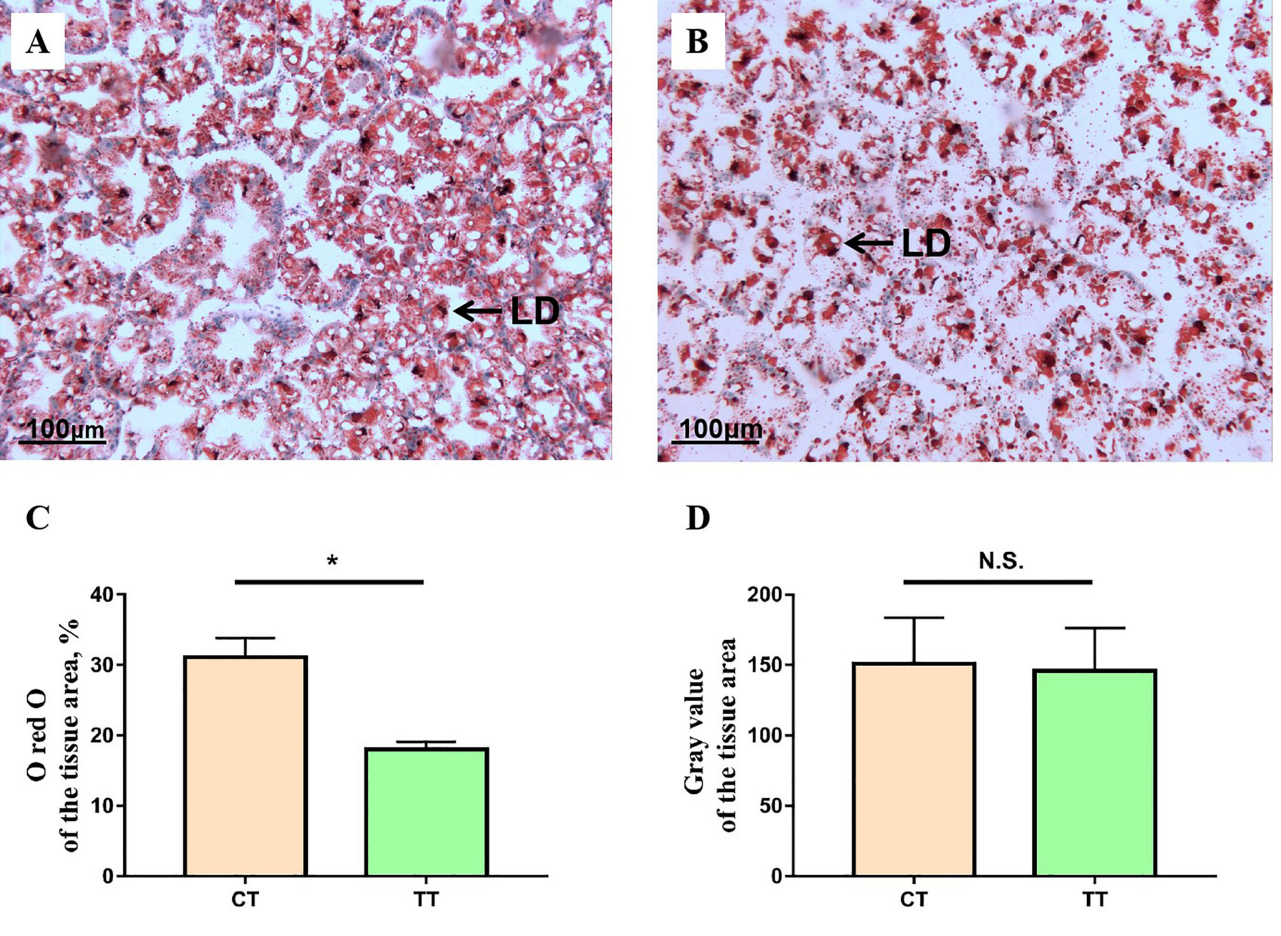
The representative micrographs of oil red O stainings. **(A)** CT group oil red O stainings; **(B)** TT group oil red O stainings, the areas stained red are lipid droplets (LD). Analysis of ORO-stained hepatopancreas lipid droplets content and gray value using the ImageJ software of two different groups **(C, D)**. Data are expressed as means with SEM. Value with an asterisk is significantly different (P < 0.05). “*” means significantly difference (P < 0.05), "n.s." means no significant difference (P > 0.05).

As shown in **Figure 2**, the 100 mg/kg TTO supplementation induced the decrease of lipid droplets in the hepatopancreas of *M. rosenbergii* (*P* < 0.05; **Figures 2A, B**). The lipids occupied almost 31.10% of the tissue area in control and were down to almost 18.03% after feeding 100 mg/kg TTO (*P* < 0.05; **Figure 2C**). Furthermore, there was no significant difference in gray value between the two groups (*P* > 0.05; **Figure 2D**).

### Hemolymph Lipid Parameters

**Figure 3:**
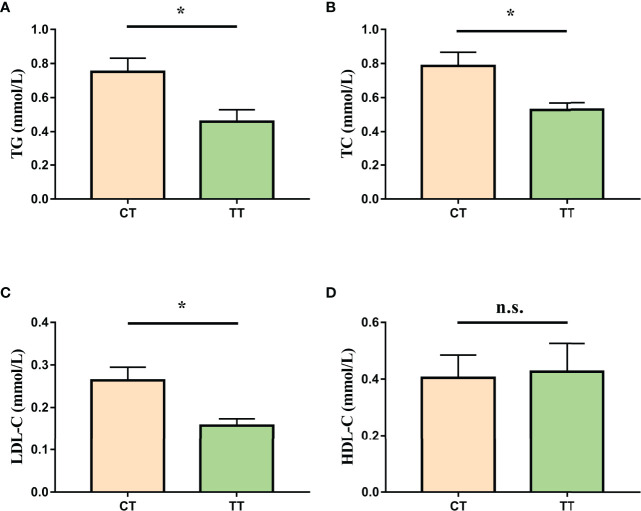
Effect of TTO on hemolymph lipid indicators in *M. rosenbergii*. **(A)** Triacylglycerol (TG); **(B)** Total Cholesterol (TC); **(C)** Low-density lipoprotein cholesterol (LDL-C); **(D)** High-density lipoprotein cholesterol (HDL-C). Data are expressed as means with SEM. Value with an asterisk is significantly different (*P* < 0.05).

To assess the effect of TTO on hemolymph lipids in *M. rosenbergii*, we investigated the levels of TG, TC, LDL-C, HDL-C. Parameters of hemolymph lipids (TG, TC, LDL-C) were significantly reduced in the TT group compared to those in the CT group (*P* < 0.05; **Figures 3A–C**). Besides, there was no significant difference in HDL-C level between the two groups (*P* > 0.05; **Figure 3D**).

### Hepatopancreas Antioxidant Enzyme Activity

**Figure 4:**
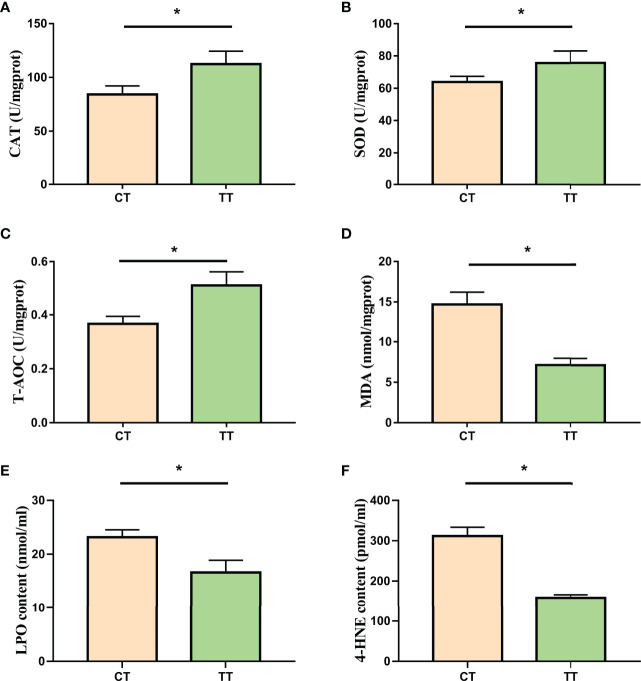
Effect of TTO on antioxidant capacity in hepatopancreas tissues of *M. rosenbergii*. **(A)** Catalase (CAT); **(B)** Superoxide dismutase (SOD); **(C)** Malonaldehyde (MDA); **(D)** Total antioxidant capacity (T-AOC); **(E)** Lipid peroxidation (LPO); **(F)** 4-hydroxynonenal (4-HNE). Data are expressed as means with SEM. Value with an asterisk is significantly different (*P* < 0.05).

The enzyme activities of T-AOC, SOD, and CAT in the TT group were significantly higher than those of the CT group (*P* < 0.05; **Figures 4A–C**), and the level of hepatopancreas MDA, LPO, and 4-HNE in the TT group was significantly lower than that of the CT group (*P* < 0.05; **Figures 4D–F**).

### Proteome Profile of the Hepatopancreas

**Figure 5:**
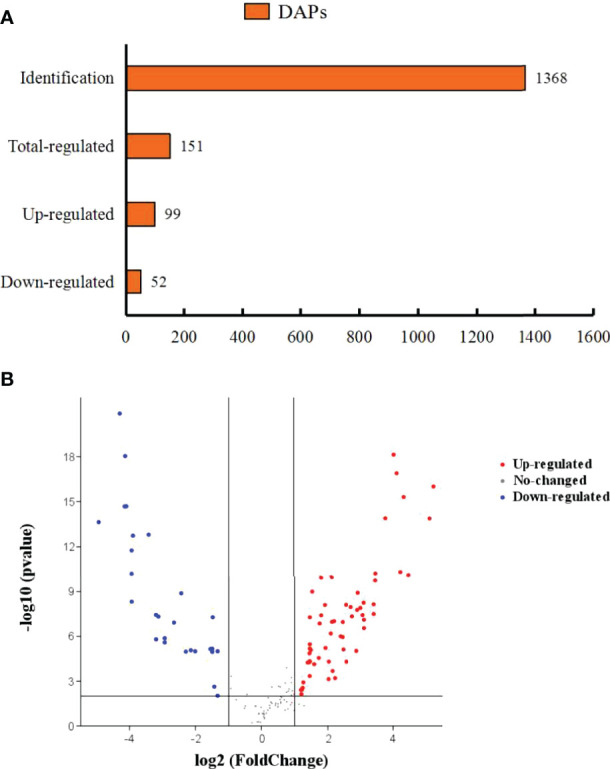
Differentially abundant proteins of *M. rosenbergii* under TTO diets. **(A)** Orange represents differentially abundant proteins. The abscissa represents quantity. **(B)** A volcano diagram shows differentially expressed proteins (DEPs) in the TT group compared to the CT group. Different colours show expression differences; red indicates up-regulation, while blue indicates down-regulation.

The number of identified proteins was 1368 (**Figure 5A**). Additionally, 151 DEPs were identified between the CT group and the TT group with at least 1.5-fold difference (**Figure 5B**). Among these DEPs, 99 DEPs were up-regulated, and 52 DEPs were down-regulated.

### Functional Analysis of the Differentially Expressed Proteins

**Figure 6:**
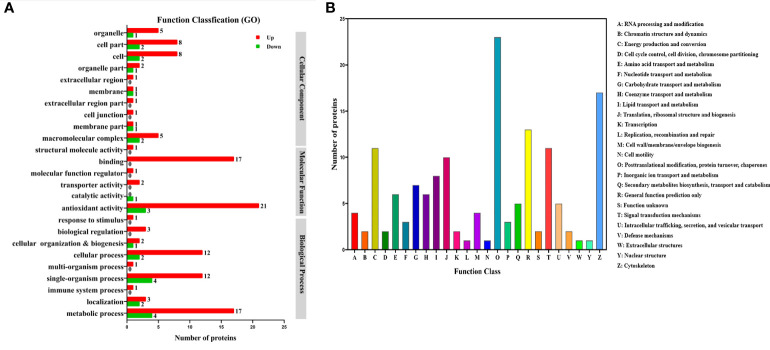
Gene Ontology (GO) **(A)** and eukaryotic ortholog Groups (KOG) **(B)** functional classification of differentially expressed proteins in the hepatopancreas of *M.rosenbergii* fed with CT and TT diets.

Function annotation of the differential expressed proteins was performed to understand the function and bioprocess involved of the differential expressed proteins. Gene Ontology (GO) annotation of the differential expressed proteins showed that both up-regulated and down-regulated proteins were mainly categorized into the metabolic process, single-organism and cellular process in biological process, catalytic activity and blinding in molecular function, and cell and cell part in cellular component (**Figure 6A**). Many proteins related to metabolism were identified according to the eukaryotic ortholog Groups (KOG) annotation of the differentially expressed proteins (**Figure 6B**). A total of 8 proteins were related to lipid transport and metabolism. The TT group also significantly changed the metabolism of proteins (7 proteins) and carbohydrates (7 proteins), respectively.

**Figure 7:**
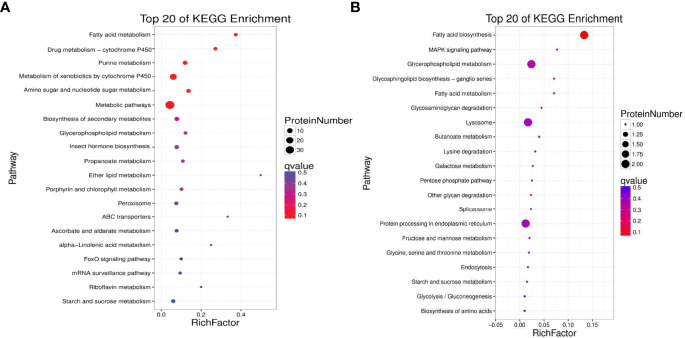
Kyoto Encyclopedia of Genes and Genomes (KEGG) enrichment analyses of up-regulated **(A)** and down-regulated **(B)** proteins between the TT group and CT group (TT vs CT). The size of the circle represents the number of DEPs. The colour represents the P-value.

Top 20 Kyoto Encyclopedia of Genes and Genomes (KEGG) pathways were annotated for the 99 up-regulated proteins in the TT group. The top five KEGG pathways were protein processing in fatty acid metabolism, drug metabolism-cytochrome P450, purine metabolism, metabolism of xenobiotics by cytochrome P450, and amino sugar and nucleotide sugar metabolism (**Figure 7A**). The corresponding DEPs were acyl-CoA oxidase 1 (ACOX1), acyl-CoA oxidase 3 (ACOX3), acetyl-CoA carboxylase (ACACA), enoyl-CoA hydratase (ECHS1), glutathione S-transferase-theta 1 (GSTT1), Glutathione peroxidase 3 (GPX3), Glutathione peroxidase 4 (GPX4), cytochrome P4501A1 (CYP1A1), cytochrome P45015A1 (CYP15A1) and heat shock 70kDa protein (HSP70), etc. 33 proteins.

KEGG annotation showed that the down-regulated 52 proteins in the TT group were affiliated to Top 20 KEGG pathways. The top five pathways were Fatty acid biosynthesis, MAPK signaling pathway, Glycerophospholipid metabolism, Glycosphingolipid biosynthesis-ganglio series, and Fatty acid metabolism(**Figure 7B**). The differentially expressed proteins that participated in these pathways were fatty acid synthase (FASN), lysophosphatidylglycerol acyltransferase 1 (LPGAT1), acetylcholinesterase (ACHE), glycerol-3-phosphate dehydrogenase 1 (Gpdh1), and Acylglycerophosphate acyltransferase 4 (AGPAT4), etc. 10 proteins.

**Figure 8:**
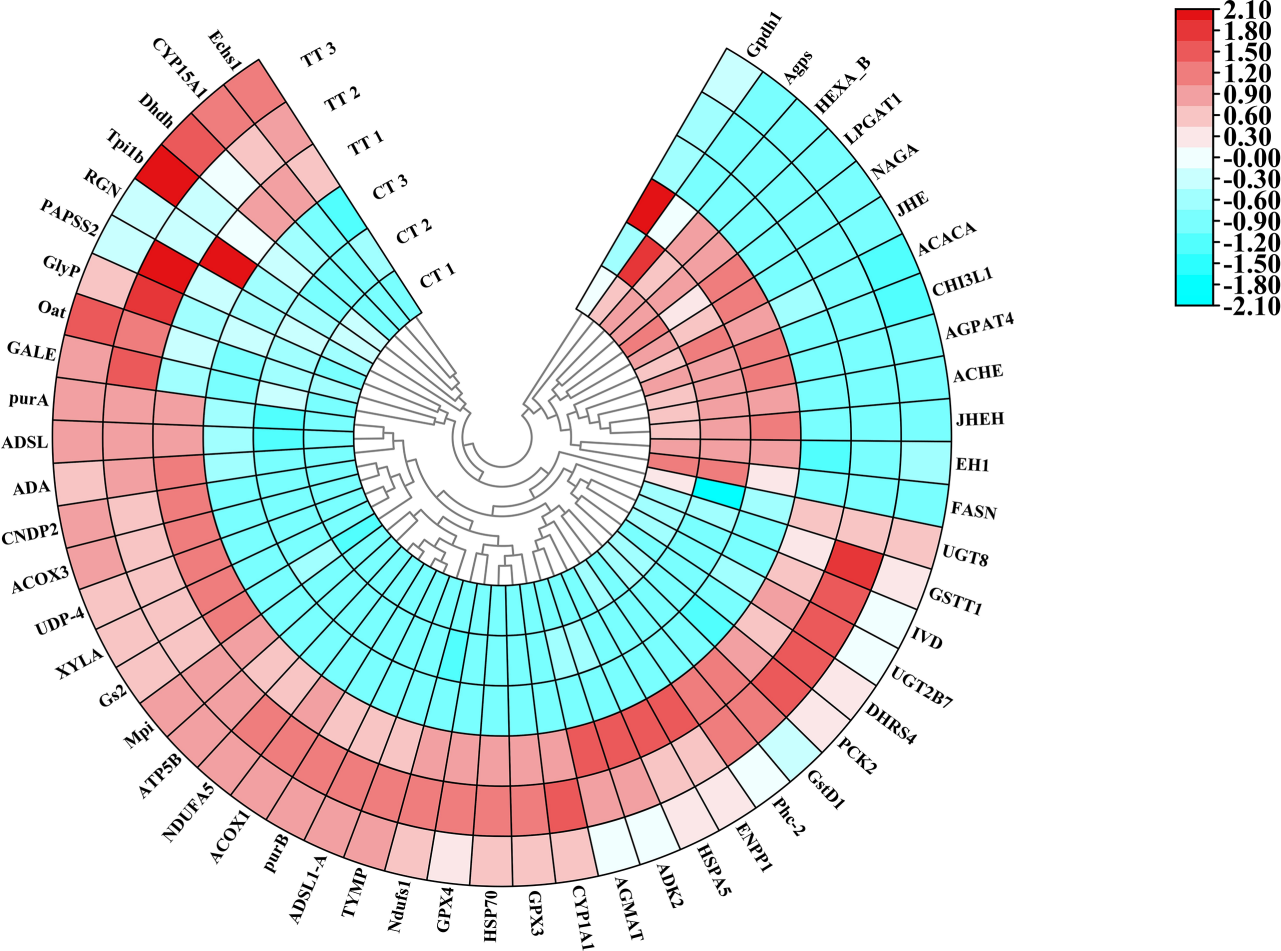
Polar Heatmap shows the expression signature of partial DEPs. Different colors show expression differences; red indicates up-regulation, while blue indicates down-regulation. Clustering analysis shows the expression signature of DEPs. CT 1, CT 2, and CT 3 represent the three replicates of the control group in protein level, TT 1, TT 2, and TT 3 represent the three replicates of the TT group in protein level.

The 54 DEPs expressions in the top five up-regulated and down-regulated KEGG pathways with CT and TT group were shown in the heat map (**Figure 8**).

### Protein-Protein Interaction (PPI) Network Analysis

**Figure 9:**
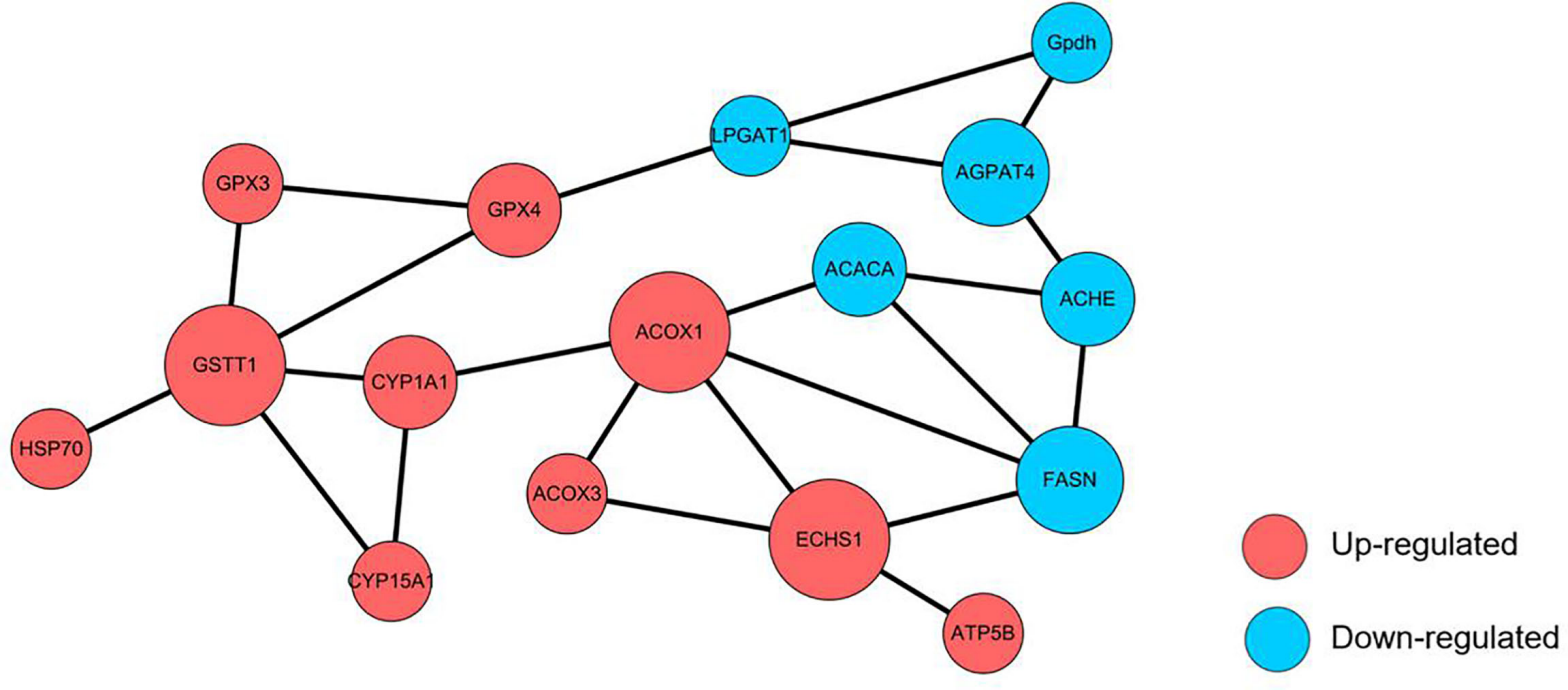
Protein-protein interaction (PPI) network analysis of altered proteins is identified in the TT group. Each node represents a protein; nodes in red and blue colour represent up-and down-regulated proteins, respectively. The size of a node represents an average of the protein abundance.

Network interaction and functional annotation analysis were performed to explore the relationship of the 54 DEPs expressions in the top five up-regulated and down-regulated KEGG pathways. The results indicated that 16 unique proteins had essential roles in the interaction network (**Figure 9**). Except for the down-regulation of AGPAT4, ACHE, FASN, GPDH, GSTT1, and LPGAT1, all other proteins were up-regulated. Among this proteins, FASN, ACACA, ECHS1, ACOX1, ACOX3, and ATP5B were functionally annotated to fatty acid synthesis and metabolism. AGPAT4, LPGAT1, GPDH, and ACHE were annotated to glycerophospholipid metabolism, and the remaining 6 proteins (CYP1A1, CYP15A1, GSTT1, GPX3, GPX4, and HSP70) were annotated to cytochrome p450 system (metabolism and antioxidant system). In addition, GPX4, LPGAT1, ACOX1, CYP1A1, ACHE, FASN and ACACA were key proteins linking different functions. These results suggested that the 16 uniquely expressed proteins could be related to enhancing the antioxidant reaction and lipid metabolism in *M. rosenbergii* hepatopancreas cells.

### Relative mRNA Expression of Altered Proteins

**Figure 10:**
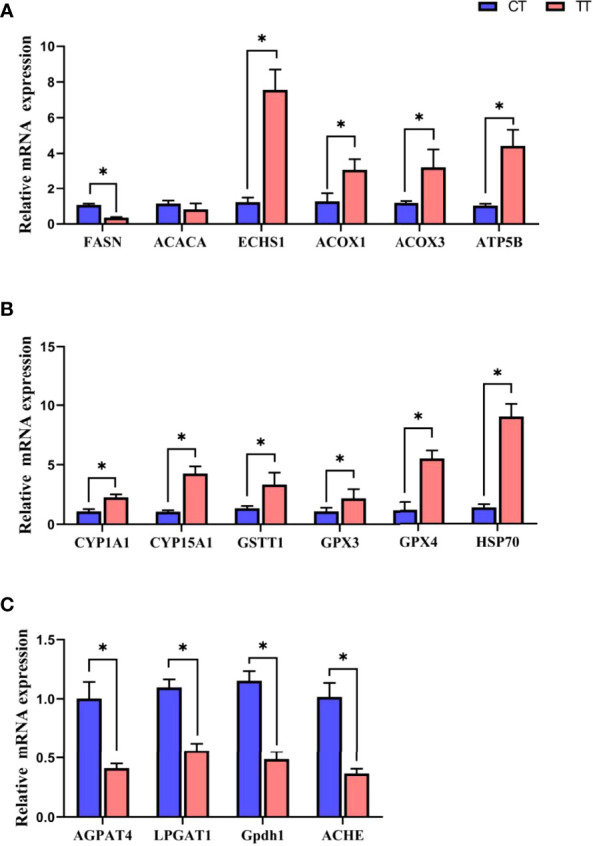
Relative mRNA expression of in Fatty acid metabolism **(A)**, Cytochrome P450 system **(B)**, and glycerophospholipid metabolism **(C)** by qRT-PCR. Data are expressed as means with SEM. Value with an asterisk is significantly different (*P* < 0.05).

16 key DEPs in PPI were validated by qRT-PCR (**Figure 10**). The mRNA expressions of 15 proteins were consistent with the trend of protein levels. Only ACACA was different trends, the ACACA proteins expression was decreased in the TT group compared with the CT group, while the ACACA did not decrease significantly (*P* > 0.05) in mRNA expression. Because PCR assays detect specific mRNAs at the transcriptional level and specific proteins at the translational level, the PCR results dominated the expression level.

### Pearson’s Correlation Analysis

**Figure 11:**
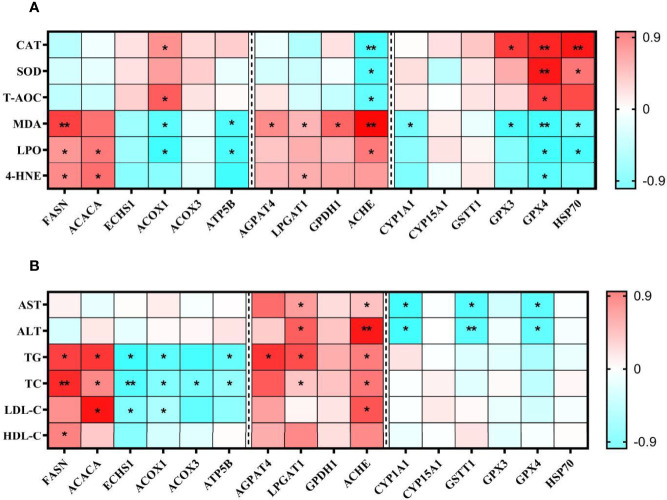
Pearson correlations of examined parameters of antioxidant capacity **(A)** and hemolymph biochemical indicators **(B)** in the TT group. In the Pearson correlations analysis, Red represents a blue correlation, negative represents a positive correlation. The darker the colour, the stronger the correlation. *P < 0.05, **P < 0.01.

**Figure 11** showed the correlation analysis of mRNA levels of DEPs with antioxidant indicators and hemolymph biochemistry parameters. The antioxidant enzymes CAT, SOD, and T-AOC activities were negatively correlated with ACHE and correlated positively with GPX4. The peroxides MDA, LPO and 4-HNE were negatively correlated with GPX4 and correlated positively with FASN. The blood lipid levels (TG, TC and LDL-C) were negatively correlated with ECHS1 and positively correlated with ACACA and ACHE. HDL-C levels were only positively correlated with FASN. The AST and ALT levels were negatively correlated with CYP1A1, GSTT1, GPX4 and positively with LPGAT1 and ACHE. The *P*-value was at least less than 0.05 when the results were correlated.

## Discussion

Although dietary essential oil could affect aquatic animals’ growth performance and health (3), most of these published reports were about fish and rarely involved in prawns. Our study found that the TT group had a better performance with the WGR, and SGR increased. He et al. (28) and Cagol et al. (29) also found that the essential oils could improve the growth performance of *M. rosenbergii* and *Litopenaeus vannamei*. In addition, the survival rate of the TT group was significantly improved compared with the CT group after ammonia nitrogen stress. Because the hepatopancreas is an essential metabolic and functional organ of crustaceans, determining growth and health. So, we speculated that these results may be attributed to tea tree oil improving liver function and health. H & E staining is a method that visually demonstrates the health of liver function. Blurred cell boundaries, densely distributed vacuoles, and tubules reduction or swollen lumen were observed, which are typical characteristics of steatosis and hepatopancreas damage (30). In our study, H & E staining results showed that the vacuoles of the hepatopancreas were decreased with clearly visible nucleus and obvious cell boundaries in the TT group compared with that of the CT group. These results revealed that 100 mg/kg TTO improved hepatopancreas health. In addition, the hepatopancreas is the main detoxification organ, so changes of hepatopancreas function and health status may result in different survival rates. Furthermore, the significantly increased AST and ALT contents in hemolymph indicated the occurrence of hepatopancreatic injury (31, 32). In our experiment, the hemolymph ALT and AST levels in the TT group were significantly lower than those in the CT group. The results suggested that TTO protected hepatopancreas health. Research on Nile tilapia also found that plant extract similar to TTO could protect liver function accompanied by AST and ALT decline (33).

In general, growth performance was positively correlated with HSI and CF when FI increased. However, in our study, we found that the FI of the TT group was higher than that of the CT group, while the HSI of the TT group decreased and CF increased significantly with the improvement of growth performance. At the same time, Oil red O-stained showed that the lipid droplets content in the hepatopancreas of the TT group was significantly reduced. Therefore, the reduction in HSI in the TT group was due to the reduction in the lipids content of the hepatopancreas, and CF increasing may be due to the development of gonadal and muscle. Previous studies have found that essential oils could promote the development of gonads (especially ovaries) and muscles (34, 35).The previous study found that Lipid is an important substrate for nutrients and the hepatopancreas is the main nutrient metabolism organ of crustaceans (8), the main function of hepatopancreatic lipid metabolism is to provide energy for growth and development of other organs (36). Based on our results, we speculate that TTO could improve the lipid metabolism of the hepatopancreas. Meanwhile, the TT group significantly reduced hemolymph TC, TG, and LDL-C levels. TC, TG, and LDL-C were all essential components of lipids (37). Similarly, the reduction in TC, TG, and LDL-C was corroborated by previous studies on treating fatty liver with plant extracts (38, 39). Maintaining liver function need better lipid metabolism capacity. Therefore, we suggested that TTO could regulate the lipid metabolism of the hepatopancreas for a protective mechanism. The mechanism of TTO regulating lipid metabolism needs to be further studied and revealed.

In recent years, the application of proteomics analysis in aquaculture has been heating up (17, 40). Based on KEGG enrichment analyses, the present study identified that TTO significantly regulated fatty acid biosynthesis and metabolism and glycerophospholipid metabolism pathways. This study found 6 different proteins (ACOX1, ACOX3, FASN, ECHS1, ACACA, and ATP5B) expressions in fatty acid biosynthesis and metabolism pathways. Combining protein expression and qPCR results, we found that the TT group down-regulated the levels of fatty acid biosynthesis related proteins (FASN, ACACA), and up-regulated the levels of oxidative catabolism of fatty acids (ECHS1, ACOX1, ACOX3, ATP5B). FASN and ACACA were two critical enzymes in fatty acid synthesis. FASN activity directly determined the ability to synthesize fatty acids, and the decreased expression level of FASN lead to a reduction in lipid deposition (41). ACACA catalyzed the first committed step in the biosynthesis of long-chain fatty acids, and triglycerides in lipids mainly were composed of multiple long-chain fatty acids (42). The FASN and ACACA levels decreased in TT group, confirming that the fatty acid synthesis capacity was decreased after TTO supplementation. Fatty acids undergo oxygen decomposition in the presence of sufficient oxygen supply, producing a large amount of energy in the body, called fatty acids β-oxidation (43). ACOX1, ACOX3, ECHS1, and ATP5B were four critical proteins involved in β-oxidation. Acyl-CoA oxidase (ACOX) families were the key rate-limiting enzymes for fatty acid β-oxidation, catalyzed the first and rate-limited reaction in β-oxidation (44). ACOX1 and ACOX3 are two specific acyl-CoA oxidases. ECHS1 could catalyze the second step in the β-oxidation (45). ATP5B is ATP synthase. It promotes fatty acid transport to mitochondria and enhance β-oxidation (46). The results representation in the fatty acid pathway reflected that TTO reduced fatty acid synthesis and promoted the oxidative decomposition of fatty acids. We also found that four proteins (AGPAT4, LPGAT1, Gpdh1, and ACHE) were down-regulated in the glycerophospholipid metabolism pathway in the TTO group compared with control. Phospholipids are generally less in body fat, and excess phospholipids can lead to obesity and liver metabolic disorders. AGPAT4, LPGAT1 and GPDH1 synthesized phosphatidic acid (PA). PA was an intermediate product in synthesizing glycerophospholipids and fatty acids (47). AGPAT4 converted lysophosphatidic acid (LPA) into PA by incorporating an acyl moiety at the sn-2 position of the glycerol backbone (48), LPGAT1 and GPDH1 were directly involved in the production of PA (49, 50). ACHE was involved in glycerol synthesis through hydrolyzing glycerophospholipids by phospholipases to lysophospholipids (51). Then, the hepatopancreas used glycerol and fatty acids as raw materials to synthesize triglycerides through the phosphatidic acid pathway (52). In addition, increased LP could damage cell membranes and red blood cells (49). Therefore, the phospholipid metabolism results suggested that TTO regulated lipid metabolism by inhibiting the production of PA and glycerol, reducing the production of harmful membrane substances to protect hepatocyte function. The above results indicated that TTO reduced the synthesis of fatty acids and glycerophospholipids and promoted fatty acid decomposition, improving lipid metabolism and protecting hepatopancreas health. Additionally, the enhanced β-oxidation in the TT group promoted the decomposition of lipids in the hepatopancreas to supply a large amount of energy for growth. This may partially explain why TTO supplementation promoted prawns growth but decreased the hepatopancreas index.

Lipid peroxides produced by lipid metabolism disorders can damage hepatopancreas health and function. In our study, The MDA, LPO, and 4-HNE decreased in the TTO group. LPO was the lipid peroxidation product, and MDA and 4-HNE were by-products generated from LPO (53, 54). As we all know, antioxidant enzyme activities were also an essential indicator for evaluating the hepatopancreas function, as these enzymes could scavenge peroxides (55). The TTO group increased the hepatopancreas antioxidant enzyme activities T-AOC, SOD, CAT in our study. These results suggested that TTO could improve the antioxidant enzyme activity to reduce peroxidative damage. Similarly, *Litopenaeus vannamei* has also reported that plant essential oils enhance antioxidant capacity, but the specific mechanism has not been elucidated (56). Superiorly, we further used proteomic analysis of the hepatopancreas on *M. rosenbergii* to find that TTO may regulate antioxidant function through the P450 pathway.

It was reported that the cytochrome P450 system was related to antioxidant defenses (57). In our current research, the 6 different proteins (CYP1A1, CYP15A1, GSTT1, GPX3, GPX4, and HSP70) we identified in the cytochrome P450 pathway were all up-regulated in the TT group. The cytochrome P450 (CYP) enzyme system was the most abundant in the liver, and various CYP enzymes perform different biological functions (58). Guengerich (59) found that the cytochrome P450 phase II enzymes played a critical role in the bioactivation and antioxidants. CYP1A1 and CYP15A1 belonged to this class of phase II enzymes and are involved in antioxidant processes (60). Phase II reactions catalyzed glucuronide and glutathione conjugate formation, respectively (61, 62). GPX3 and GPX4 were two different glutathione peroxidases, which catalyzed the reduction of hydrogen peroxide, lipid peroxides, and organic hydroperoxides by glutathione, protecting cells from oxidative damage (63, 64). GSTT1 was a kind of glutathione transferase, mainly catalyzing the covalent combination of peroxides and their metabolites with the sulfhydryl group of glutathione to achieve the purpose degradation (65). Besides, HSP70 could inhibit NADH enzyme activity (a key enzyme for free radical production) and reduce free radical production (66). The results of up-regulation expression suggested that TTO regulated antioxidant enzyme activity in the cytochrome P450 system to achieve the purpose of anti-oxidation.

Previous research has shown that fatty acid metabolism, glycerophospholipid metabolism, and the cytochrome P450 enzyme system were interconnected (67). Our PPI network analysis results showed that phospholipid metabolism-related protein ACHE interacted with lipid metabolism-related proteins ACACA and FASN. Previous studies showed that inhibition of ACHE suppressed FASN and ACACA expression, ultimately leading to lipid homeostasis (68). ACHE could degrade acetylcholine (ACH), and ACH deficiency caused metabolic syndrome and led to fat metabolism disorder, which was also the source of lipid increase (69). Therefore, when ACHE was inhibited, sufficient acetylcholine could be provided to prevent overexpression of FASN and ACACA from maintaining a steady state of lipid metabolism. Furthermore, the ACOX1 protein in the fatty acid metabolism pathway interacts with the CYP1A1 protein in the cytochrome p450 pathway in this study. Both ACOX1 and CYP1A1 are the target genes of peroxisome proliferator-activated receptor alpha (PPARα) (70), and the activation of PPARα up-regulates the expression of a large number of peroxidase and fatty acid transport genes, resulting in lipid-lowering and antioxidant effects (71). Besides, GPX4 in the p450 pathway interacts with LPGAT1 in the glycerophospholipid metabolic pathway after TTO treatment in this study. Excessive production of phosphatidate (LPGAT1 participates in synthesis) increased phospholipid hydroxides, which were toxic to cell membranes (72). GPX4 was the only enzyme identified thus far that acts on phospholipid hydroperoxides and activation of GPX4 inhibit phospholipid hydroxide production (73). Its upregulation expression directly explained the reasons for the enhanced antioxidant and decreased hepatopancreatic cytotoxicity in the TT group. Therefore, the protein interaction mechanism in the pathways plays a crucial role in improving antioxidant capacity, lipid metabolism, and hepatopancreatic health after TTO addition.

The results of Pearson’s correlations reinforced the previous hypothesis in this study. Most of the analyses are consistent with our previous conclusions, but it is worth noting that something did not demonstrate. Fatty acid and glycerophospholipid synthesis proteins (FASN, ACACA, AGPAT4, LPGAT1, GPDH1, ACHE) positively correlated with lipid peroxides, whereas fatty acid metabolizing proteins (ACOX1 and ATP5B) have opposite results. Fatty acid and glycerophospholipid synthesis proteins are involved in fat synthesis, and their upregulation leads to increased fat synthesis. It has been reported that excessive accumulation of liver fat can cause damage to hepatopancreatic cells caused by harmful lipid peroxides combined with oxygen free radicals (74). In addition, free radicals react with cell membrane phospholipids (glycerophospholipid synthesis) to produce lipid peroxides (75). Studies have demonstrated that the downregulation of these proteins reduces lipid peroxidation activity (76, 77). Besides, CYP1A1 and GSTT1 were inversely correlated with the AST and ALT. Some enzymes in the CYP family could promote the conversion of toxins into non-toxic or low-toxic metabolites in the body (65), CYP1A1 was one of these enzymes. GSTT1 could catalyze the degradation of toxic substances which performs similar functions as CYP1A1 (78). The down-regulated CYP1A1 and GSTT1 indicate that the hepatopancreas’ detoxification ability is enhanced after TTO supplementation.

At last, combined with the growth evaluation, we speculate that the enhanced growth performance after TTO supplementation was due to the promoting gonad growth. Because the study in crustaceans has shown that the improvement of lipid metabolism in the hepatopancreas can transfer lipids to the ovary to promote ovarian development (79). Meanwhile, affecting glycerophospholipid metabolism could also promote ovarian development in shrimp (80), and our results also found the TTO regulated glycerophospholipid metabolism. In addition, studies have found that CYP15A1 could catalyzed epoxidation of methyl farnesoate to juvenile hormone (JH), and JN could promote the development and growth of the adult ovary (81). At the same time, we also found in **Figure 8** that the TT group down-regulated the expression of juvenile hormone esterase (JHE) and juvenile hormone epoxide hydrolase (JHEH) proteins. JHE and JHEH could degrade JH (82), so the decrease of JHE and JHEH means the JH was increased in the body. Prawns and insects are very homologous species, so the results of CYP15A1, JHE and JHEH also verified our speculation for the enhanced growth performance by ovarian development of *M. rosenbergii* after TTO supplement from another angle. These provides evidences that the TT group improves the hepatopancreatic lipid metabolism, and increases the growth performance.

To sum up, TTO could reduce the lipid peroxides induced damage to hepatopancreas cells, and exert a protective effect on the hepatopancreas. The reduction of lipid peroxides is mediated by the targeted regulation of critical enzymes in the lipid metabolism process and the antioxidant reaction of TTO to reduce the generation of oxygen free radicals in the lipid metabolism process. In addition, TTO may enhancing growth performance by promoting hepatopancreatic lipid transfer to the gonad. Therefore, TTO may have a potential preventive effect on hepatopancreatic cell damage caused by lipid metabolism disorder and improve growth performance. It is necessary to make more detailed studies to further assess the possible relationships between antioxidants and lipid metabolism.

## Data Availability Statement

The datasets presented in this study can be found in online repositories. The names of the repository/repositories and accession number(s) can be found below: ProteomeXchange, accession ID: PXD032896.

## Author Contributions

ML, PX, and BL (8th author) contributed to conception and design of the study. XZ organized the proteomic database. CS and QZ performed the statistical analysis. ML wrote the first draft of the manuscript. All authors contributed to manuscript revision, read, and approved the submitted version.

## Funding

This work was supported by the Project of National Key R&D Program of China (2019YFD0900200), China Agriculture Research System of MOF and MARA (CARS-48), the National Natural Science Foundation of China (32002404), Central Public-interest Scientific Institution Basal Research Fund, CAFS (2020TD59). The authors would like to express their sincere thanks to the personnel of these teams for their kind assistance.

## Conflict of Interest

The authors declare that the research was conducted in the absence of any commercial or financial relationships that could be construed as a potential conflict of interest.

## Publisher’s Note

All claims expressed in this article are solely those of the authors and do not necessarily represent those of their affiliated organizations, or those of the publisher, the editors and the reviewers. Any product that may be evaluated in this article, or claim that may be made by its manufacturer, is not guaranteed or endorsed by the publisher.
