# Supplementary figures and images for: Comparative Proteomic Analysis Revealed the Mechanism of Tea Tree Oil Targeting Lipid Metabolism and Antioxidant System to Protect Hepatopancreatic Health in Macrobrachium rosenbergii
Source: Front Immunol. 2022 May 31;13:906435. doi: 10.3389/fimmu.2022.906435 (PMC9195101; doi:10.3389/fimmu.2022.906435)

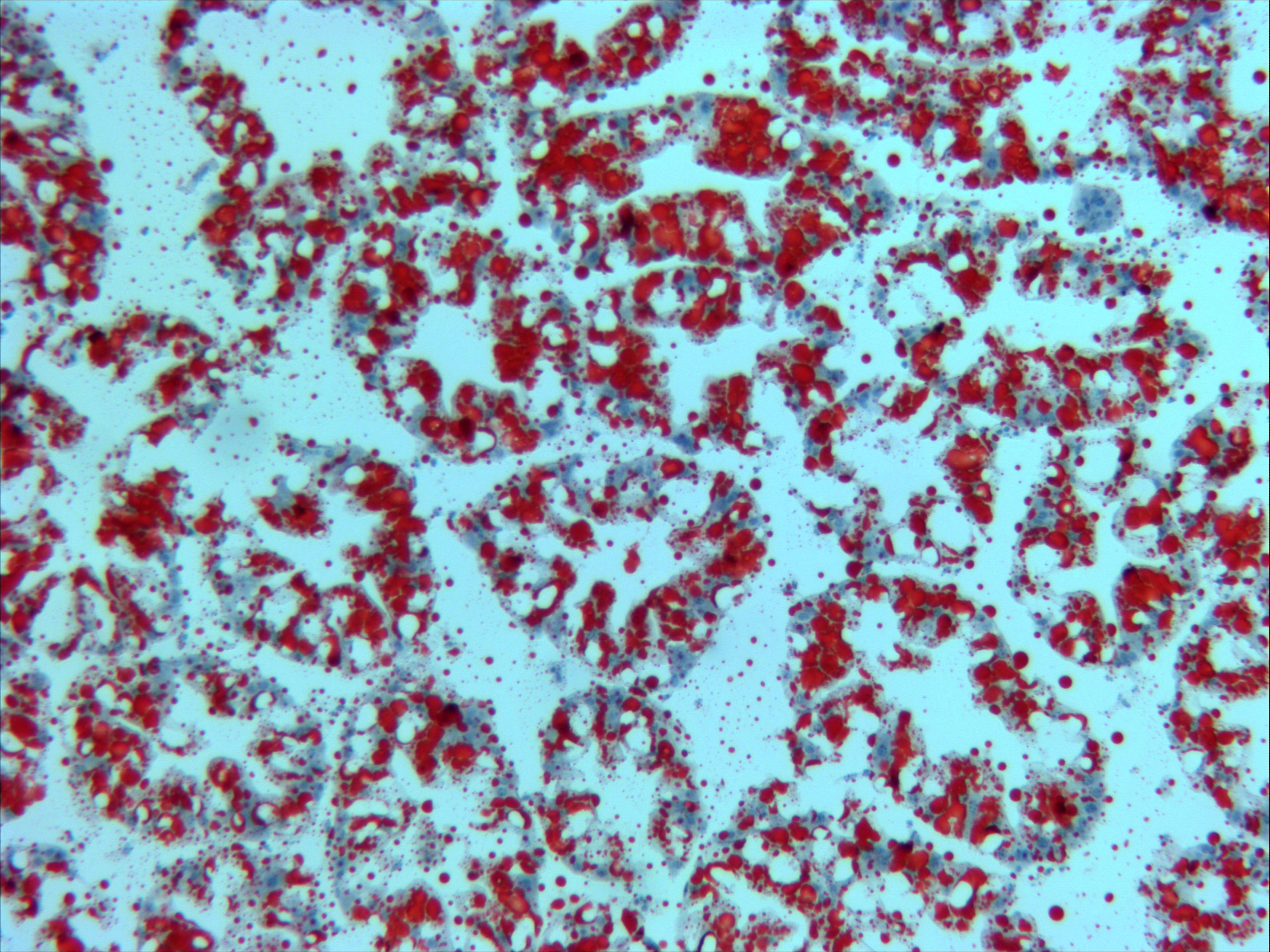

Supplement: Supplementary file 2 [file Image_1.jpeg]

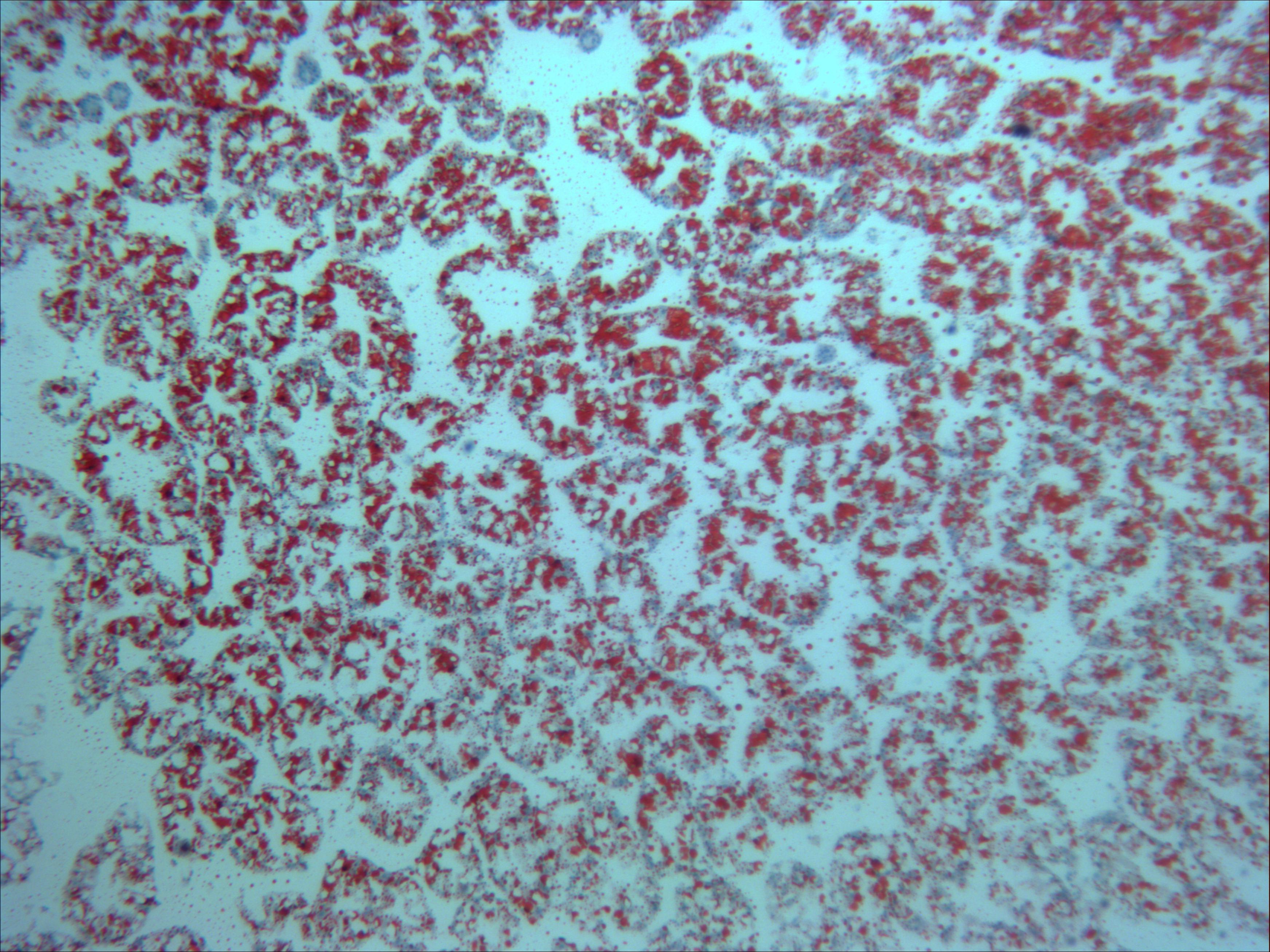

Supplement: Supplementary file 3 [file Image_2.jpeg]

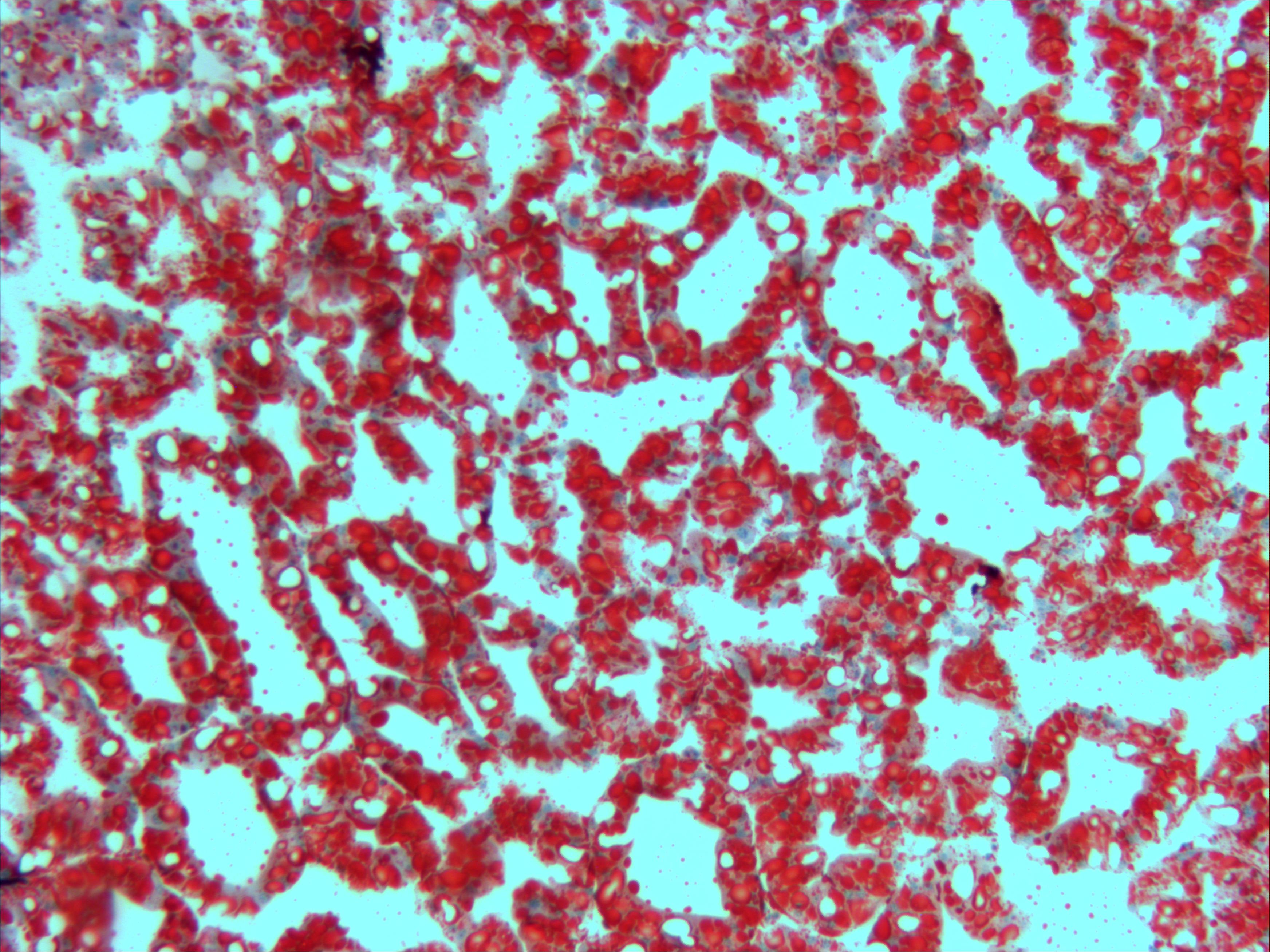

Supplement: Supplementary file 4 [file Image_3.jpeg]

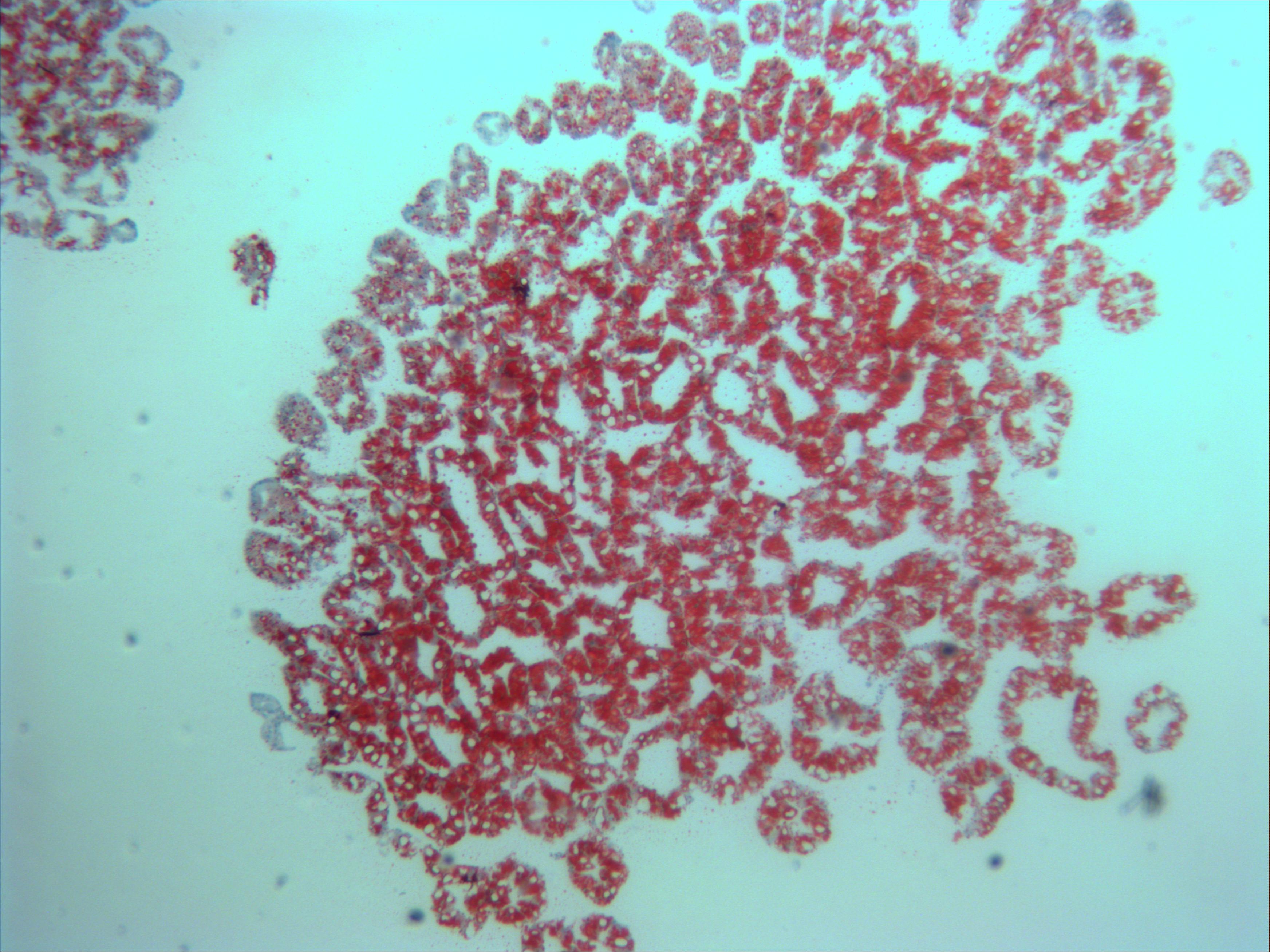

Supplement: Supplementary file 5 [file Image_4.jpeg]

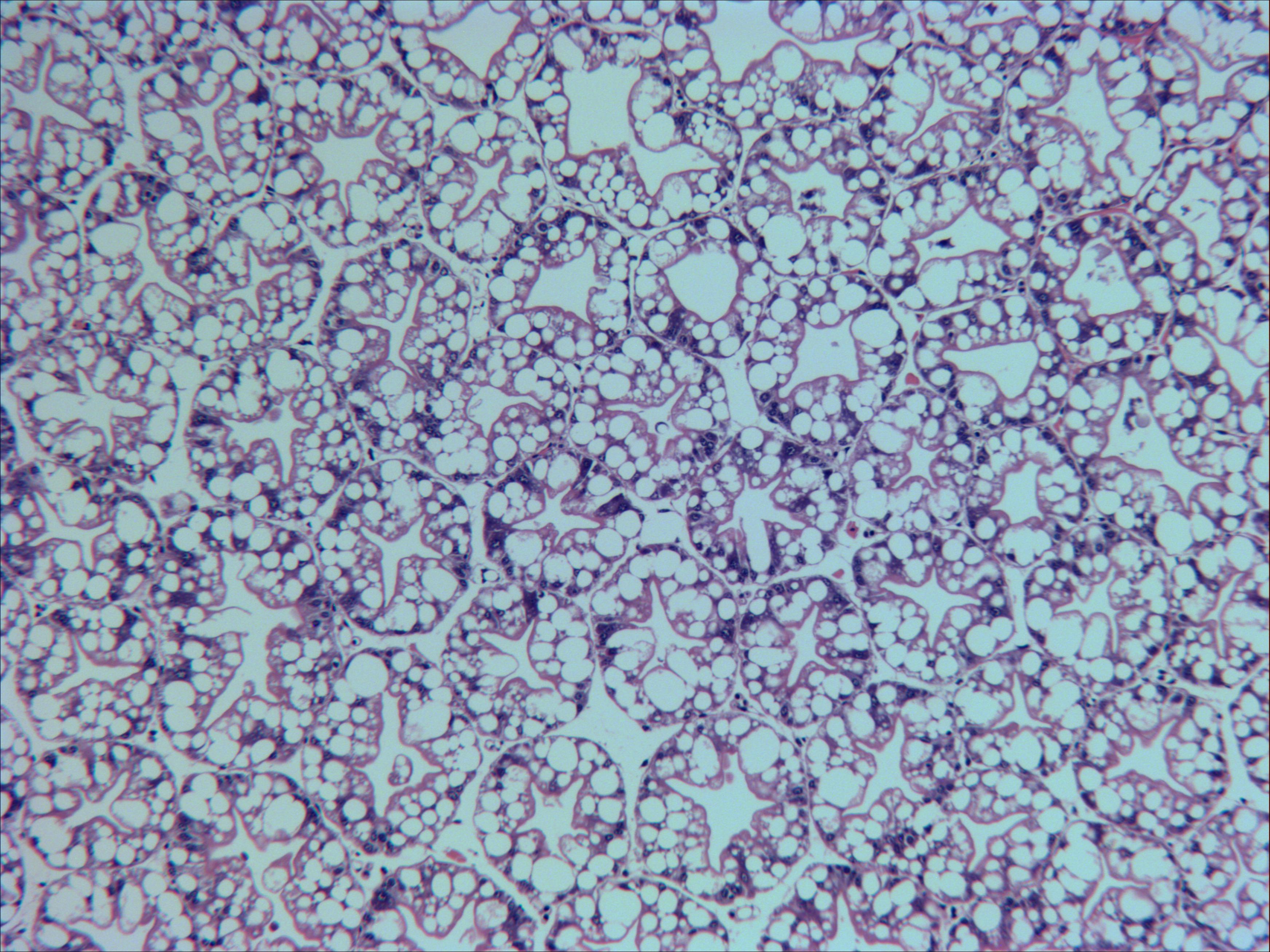

Supplement: Supplementary file 6 [file Image_5.jpeg]

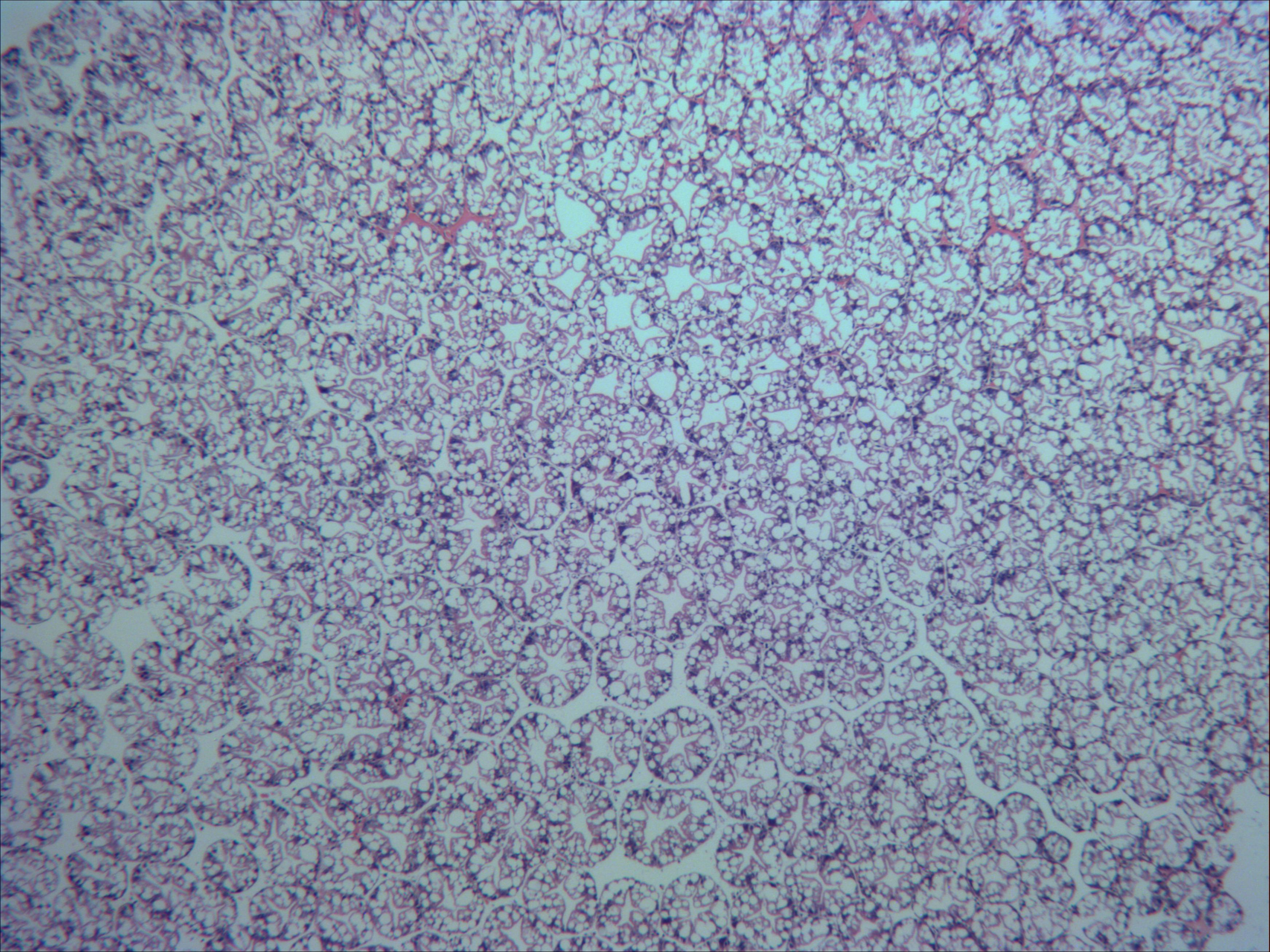

Supplement: Supplementary file 7 [file Image_6.jpeg]

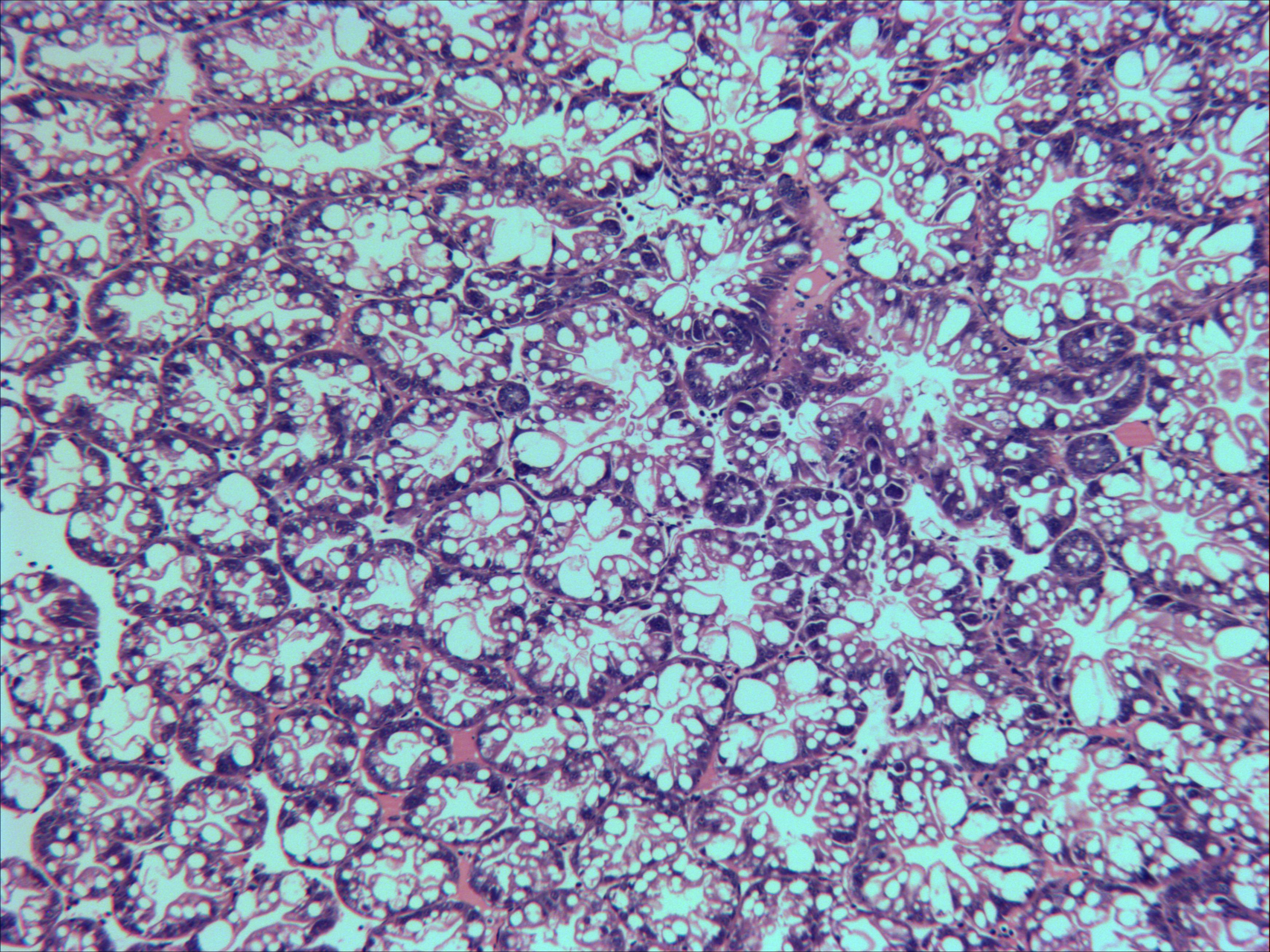

Supplement: Supplementary file 8 [file Image_7.jpeg]

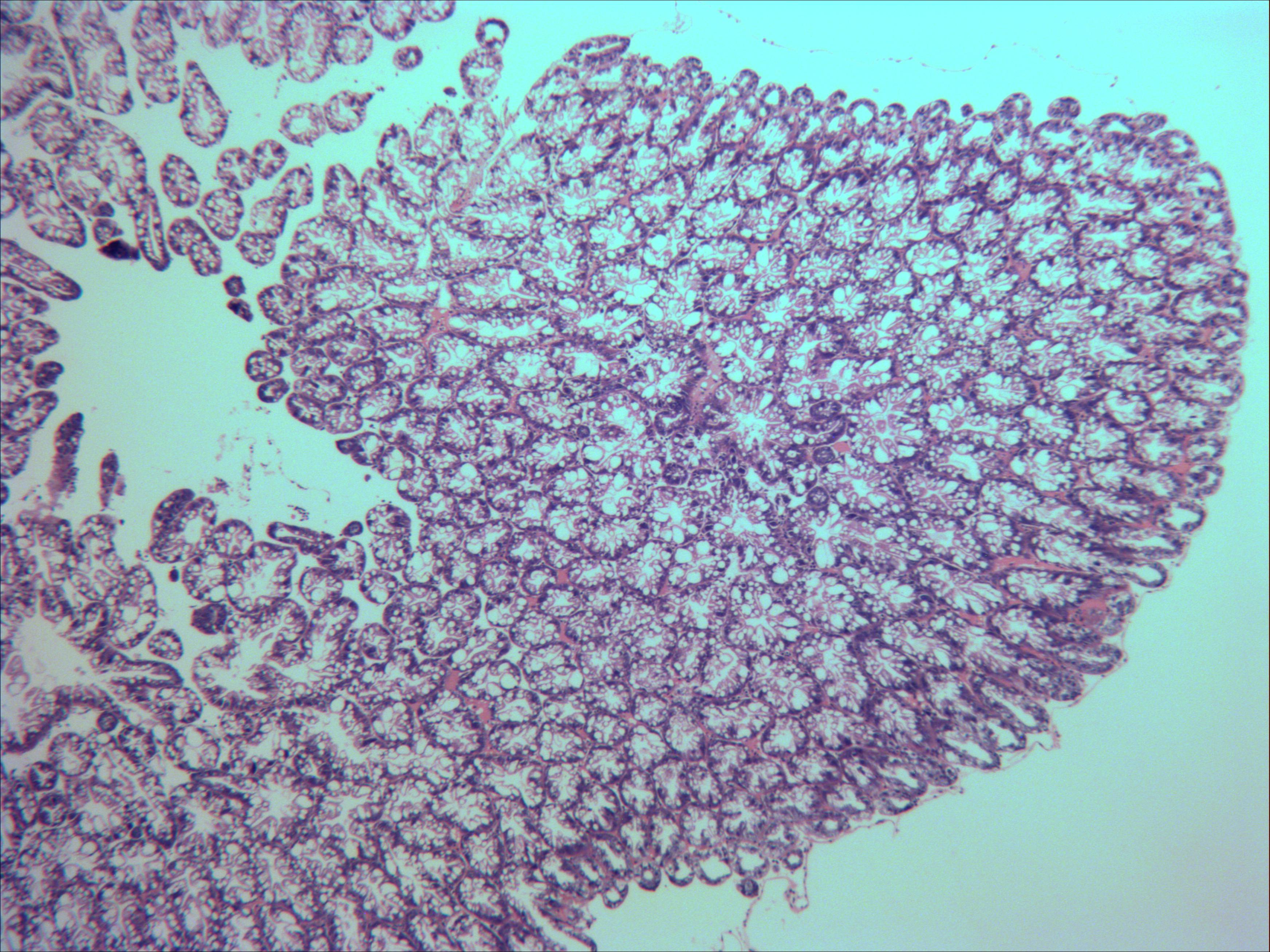

Supplement: Supplementary file 9 [file Image_8.jpeg]
